# Two novel strains isolated from a brackish lake constitute *Aeoliella fuhlenseeensis* sp. nov. and *Bythopirellula superficialaquae* sp. nov. in the family *Lacipirellulaceae*

**DOI:** 10.1038/s41598-026-59682-6

**Published:** 2026-06-29

**Authors:** Nicolai Kallscheuer, Gaurav Kumar, Jonathan Hammer, Tom Haufschild, Christian Jogler

**Affiliations:** 1 Department of Microbial Interactions, Institute for Microbiology, Friedrich Schiller University, Jena, Germany; 2 Cluster of Excellence Balance of the Microverse, Friedrich Schiller University, Jena, Germany

**Keywords:** *Planctomycetota*, Brackish strains, Schlesner strain collection, *Bythopirellula goksoeyrii*, Computational biology and bioinformatics, Evolution, Genetics, Microbiology

## Abstract

The strain collection of Heinz Schlesner (Kiel University, Germany) largely consists of budding bacteria collected from the 1980s to early 2000s. It still holds an untapped diversity of bacteria with uncommon morphologies and cell division modes. Here, we characterize two strains from his collection, SH292^T^ and SH678^T^, that he isolated from Lake Fuhlensee, a slightly brackish lake in Northern Germany. Phylogenetic tree reconstruction places the novel strains in the family *Lacipirellulaceae* within the phylum *Planctomycetota*. More detailed analyses based on five phylogenetic markers suggest positions within the genera *Aeoliella* and *Bythopirellula*, respectively. The novel isolates have an aerobic and heterotrophic lifestyle and a mesophilic and neutrophilic growth profile. Optimal growth was observed at 25 °C and pH 7.0–7.5. Genome sequencing revealed slightly smaller genomes of 5.6–6.0 Mbp compared to those of the close relatives (6.1–7.8 Mbp). An analysis of genome-encoded features suggests differences in the portfolio of secondary metabolite-associated biosynthetic gene clusters and carbohydrate-active enzymes. Genomic and morphological differences support the results of the phylogenetic inferences that together delineate the novel strains from previously described species. Hence, we conclude that the two novel isolates belong to two novel species, for which we introduce the names *Aeoliella fuhlenseeensis* sp. nov. and *Bythopirellula superficialaquae* sp. nov. The novel taxa are represented by strains SH292^T^ (= DSM 116587^T^ = KCTC 102092^T^) and SH678^T^ (= DSM 116729^T^ = KCTC 102079^T^) as the corresponding type strains.

**Supplementary Information:**

The online version contains supplementary material available at 10.1038/s41598-026-59682-6.

## Introduction

The bacterial phylum *Planctomycetota* comprises a diverse and ecologically vital group distinguished by uncommon cellular traits and intricate life cycles. Strains belonging to the phylum are ubiquitous across marine, freshwater, and terrestrial habitats and are frequently captured, both in cultivation-independent 16S rRNA gene amplicon or metagenome studies, and in cultivation-dependent studies that focus on the isolation of novel strains^1–4^. To date, the phylum is constituted by the two validly described classes *Planctomycetia*^5^ and *Phycisphaerae*^6^ and the two provisional classes ‘*Candidatus* Brocadiia’ and ‘*Candidatus* Uabimicrobiia’^7^, each with their ecological and cell biological peculiarities^2^. Members of the ‘*Ca.* Brocadiia’ class perform anaerobic ammonium oxidation (anammox), a process driving 30–50% of nitrogen loss in oxygen-depleted marine environments^8^. The class ‘*Ca*. Uabimicrobiia’ currently includes only two reported provisional species that are called bacteria of prey (BoP)^9,10^. These BoPs may have important evolutionary implications due to their obligatory predatory lifestyle involving uptake of prey bacteria by an uncharacterized phagocytosis-like mechanism.

In general, planctomycetotal bacteria contribute to carbon cycling by breaking down complex carbohydrates, including decorated polysaccharides derived from aquatic phototrophs^11^. This process involves a yet uncharacterized uptake system in combination with a set of carbohydrate-active enzymes (CAZymes) and accessory enzymes^12–14^. The heterotrophic lifestyle aligns with their frequent association with eukaryotic hosts, such as sea grasses, green and red algae, sponges, jellyfish and termites where they can account for up to 80% of the microbial community^15–30^. Planctomycetes are also linked to cyanobacterial blooms and adhere to both biotic and abiotic submerged surfaces^4,31,32^. Remarkably, despite their preference for mild temperature conditions (typical temperature optima of 24–30 °C), some strains have been isolated from extreme environments like submarine volcanic sites^33–35^.

Unlike typical Gram-stain-negative bacteria, planctomycetes exhibit distinct cell biological characteristics, including extensive internal membrane structures, condensed DNA, and asymmetric cell division through ‘budding’^2,13^. Some species possess enigmatic subcellular structures yet to be fully characterized^36^. While peptidoglycan is present in their cell walls^37,38^, certain essential genes for its synthesis, such as those in *Planctopirus limnophila*, turned out to be dispensable^2,39^. Notably, a homologue of the gene encoding the Z ring-forming FtsZ protein, critical for binary fission in most bacteria, cannot be found in the genomes of planctomycetes, highlighting their unconventional division mechanism^39,40^.

Recent research has also highlighted the role of planctomycetes as a promising source of bioactive molecules. While earlier studies relied on genome mining and activity assays in cell extracts, the past five years have seen the elucidation of specific chemical structures^41–47^. Advances in the development of genetic tools have facilitated these discoveries by enabling gene function studies through deletion mutants and the expression of genes encoding reporter proteins^48–50^.

Despite their ecological and expected future biotechnological importance, the diversity within the phylum *Planctomycetota* remains largely uncharted, with numerous lineages yet to be explored. The current collection of axenic cultures includes approximately 150 strains. This study introduces two novel members of the family *Lacipirellulaceae* (order *Pirellulales*, class *Planctomycetia*) that currently includes eight described genera and 14 described species^4,15,24,51–54^. The family was formally described in 2020 based on the type genus *Lacipirellula*, with *Lacipirellula parvula* as the type species, to accommodate a distinct phylogenetic lineage of *Pirellula*-like planctomycetes^53^. The type strains of the currently described species have been isolated from limnic or marine habitats: surface waters, deep sea sediments or from the surfaces of macroscopic phototrophs or other biotic or abiotic surfaces^4,51,53,54^. The genera *Aeoliella* and *Bythopirellula*, to which the novel isolates belong, are currently constituted by two species each^51,52,54^. Except for *Bythopirellula goeksoyrii*, that was described in 2013, the current taxa have been described in the last five years. *B. goksoeyrii* Pr1d is one of the rare examples of planctomycetotal strains that have been isolated from the deep sea^54^. Despite dwelling in this extreme environment, the lifestyle of the strain was comparable to a close relative isolated from surface waters^55^. This exciting finding requires future attention but more isolates from deep sea environments are required for allowing reliable conclusions.

The here described novel isolates belong to the strain collection of Heinz Schlesner, a researcher on budding bacteria at Kiel University. Schlesner’s work, spanning Northern Germany and international collaborations in the late twentieth century, led to the isolation of hundreds of novel strains and the characterization of foundational planctomycete species, including *Pirellula staleyi*^56^, *Blastopirellula marina*^57^, *Rubinisphaera* *brasiliensis*^58^, and *Rhodopirellula baltica*^59^, the latter being the first planctomycete with a sequenced genome^60^.

## Materials and methods

### Sampling, isolation of strains and 16S rRNA gene sequencing

The two characterized strains, SH292^T^ and SH678^T^, were both isolated from surface water of Lake Fuhlensee close to Kiel, Northern Germany (exact location: 54.431700, 10.161246). The brackish lake has a salinity in the range of 1.2–1.8% and is part of a nature reserve with a characteristic surrounding vegetation consisting of an alder swamp forest and fen meadows.

The two isolates were inoculated in M13(3x) medium (SH292^T^) or M31PY medium (SH678^T^) from the cryo stocks prepared by Heinz Schlesner. Media were prepared as previously described^61^. The addition “PY” indicates that the media contained 0.25 g/L peptone and 0.25 g/L yeast extract. The “3X” designation indicates that the medium contains three times the amounts of peptone, glucose, and yeast extract recommended for the standard M13a medium. Both strains grew on plates solidified with 15 g/L agar or in liquid medium. The 16S rRNA gene of the isolates was amplified by PCR, purified based on a standardized workflow^62,^ and sequenced at Macrogen Europe (Amsterdam, The Netherlands).

### Physiological analyses

For determination of the temperature optimum for growth, 100 µL of an exponentially growing culture was plated on the respective media mentioned above. Plates were incubated in triplicates at temperatures of 4, 10, 18, 21, 24, 28, 32, 37 and 42 °C as previously described^63^. The temperature at which bacterial lawn/colonies appeared the earliest was considered the temperature optimum for growth. The pH optimum for growth was determined in liquid cultures with 100 mM of the following buffering agents: 2-(*N*-morpholino)ethanesulfonic acid (MES) for pH 5.0 and 6.0, 4-(2-hydroxyethyl)-1-piperazineethanesulfonic acid (HEPES) for pH 7.0, 7.5 and 8.0 or *N*-cyclohexyl-2-aminoethanesulfonic acid (CHES) for pH 9.0 and 10.0. Growth was evaluated by measuring the optical density at 600 nm (OD_600_). The strains were incubated in a BioTek Epoch2 microplate spectrophotometer (Agilent, Waldbronn, Germany) with constant shaking over a cultivation time of two weeks at the determined optimal temperature for growth. One measurement cycle lasted 30 min in total and consisted of two shaking periods interrupted by the measurement of the whole 96-well plate (Brand Plate pureGrade™ S, transparent sterile 96-well plates) (Brand, Wertheim, Germany). To prevent condensation, the lid temperature was maintained at 2 °C above the incubation temperature. Obtained data points (from duplicate measurements) were corrected by subtraction of the OD_600_ of the medium blanks. The growth rate at each tested pH value was obtained from the maximal slope after plotting the ln(OD_600_) against the cultivation time.

### Microscopy and cell size determination

Light microscopy was conducted as previously described^63^. Briefly, cells from liquid cultures at the mid-exponential phase were mounted on a 1% agarose cushion (w/v, dissolved in dH_2_O). After drying at room temperature, a cover slip was placed on top and the edges were fixed with VLAP (33% vaseline, 33% lanoline, 33% paraffin, w/w) to prevent the cover slip from moving. Cells were imaged in an inverse Nikon Ti2 microscope equipped with a Nikon Plan Apo λ 100 × immersion oil objective with a phase ring for PhC images and without a phase ring for DIC images, a Nikon DS-Ri2 camera, and the NIS-Elements software (version 5.30) (Nikon, Amstelveen, The Netherlands). Cell sizes were determined as previously described^64^.

### Genome sequencing and assembly

Isolation of genomic DNA as well as genome sequencing (using Oxford Nanopore and Illumina sequencing platforms), assembly and polishing were performed as previously described^63^ with the modifications reported elsewhere^65^. Parts of the bioinformatic workflow were performed on the Galaxy web-based platform and the server available under the public domain usegalaxy.eu^66^ as previously reported^63^. Details on the sequencing chemistry and bioinformatic tools including tool version numbers and operational parameters are stated in Supplementary Table S1. Genome completeness, coding density and DNA G + C content were evaluated using CheckM version 1.2.3 with marker set “Planctomycetes” ^67^. Following initial annotation with Prokka v. 1.15.6^68^, the chromosome was re-oriented to the start codon of the *dnaA* gene, encoding the replication initiator protein, and subjected to final re-annotation using PGAP (version 2025-05-06, build 7983)^69^.

### Nucleotide sequence accession numbers

The 16S rRNA gene sequences were deposited in the GenBank database under the accession numbers PV984964 (SH292^T^) and PV984966 (SH678^T^). Genome sequence information is available from NCBI under the following accession numbers: CP197417 (SH292^T^), CP197426 (SH678^T^, chromosome), CP197427 (plasmid pSH678_1), CP197428 (plasmid pSH678_2), CP197429 (plasmid pSH678_3), and CP197430 (plasmid pSH678_4).

### Phylogenetic and genome-based analyses

The full-length 16S rRNA gene sequences of the novel isolates were extracted from the prokka-annotated genomes and were used for the identification of the current closest relatives using NCBI BLAST. Maximum-likelihood phylogenetic trees based on 16S rRNA gene sequences and multi-locus sequence analysis (MLSA) were computed for the novel strains and the described type strains of all species in the current class *Planctomycetia* as of January 2026. The MLSA-based tree only included members of the family *Lacipirellulaceae* and the outgroup. NCBI RefSeq accession numbers of the used genomes are provided in Table S2. The 16S rRNA gene sequences of *Opitutus terrae* PB90-1^T^ (NCBI acc. no. AJ229235), *Kiritimatiella glycovorans* L21-Fru-AB^T^ (acc. no. NR_146840) and *Lentisphaera araneosa* HTCC 2155^T^ (accession number NR_027571) (members of the *Planctomycetota-Verrucomicrobiota-Chlamydiota* (PVC) superphylum outside of the phylum *Planctomycetota*) served as outgroup. Sequence alignments were performed with ClustalW^70^ and IQ-TREE v.3.0.1^71^ was used for tree reconstruction with ModelFinder und 1000 ultrafast bootstrap replications. The MLSA-based phylogeny was computed with autoMLST (automlst-simplified-wrapper tool) based on 30 single copy gene-encoded proteins with 1000 bootstrap replicates^72^ and the genomes of *Rhodopirellula baltica* SH1^T^ (GenBank acc. no. BX119912.1), *Pirellula staleyi* DSM 6068^T^ (acc. no. CP001848.1) and *Blastopirellula marina* DSM 3645^T^ (acc. no. GCA_000153105.1) (all belonging to the family *Pirellulaceae*) as outgroup. Phylogenetic trees were visualized with iTOL v6^73^. The 16S rRNA gene sequence similarity matrix was obtained with TaxonDC^74^ based on the ClustalW alignment that was also used for the construction of the phylogenetic tree. Average amino acid identities (AAI) and average nucleotide identities (ANI) were calculated using respective scripts of the enveomics v.1.10.0 collection (ANI was calculated as ANIb using BLAST)^75,76^. Additional phylogenetic markers, i.e. *rpoB* sequence similarity and percentage of conserved proteins (POCP), were calculated as described^77,78^. The pangenome of selected strains was generated with anvi’o v.8^79^ with the following default parameters: minimum minbit value of 0.5, MCL (Markov Clustering) inflation of 2 and minimum amino acid sequence identity of 0%. The “Estimate SCG taxonomy” function of anvi’o v.8 was used to classify the novel isolates based on the GTDB taxonomy^80^. Biosynthetic gene clusters (BGCs) were predicted with antiSMASH v.8.0^81^ in relaxed mode and with all extra features activated. Carbohydrate-active enzymes (CAZymes) were analyzed with dbCAN3^82^; for CAZyme domain/family annotation via HMMER an e-value of 1e-15 and a coverage cutoff value of 0.35 were used. For the analysis of pangenome, GTDB taxonomy, BGCs and CAZyme-encoding genes, the non-annotated raw genome fasta files were used as input.

## Results and discussion

### Phylogenetic inference

**Fig. 1:**
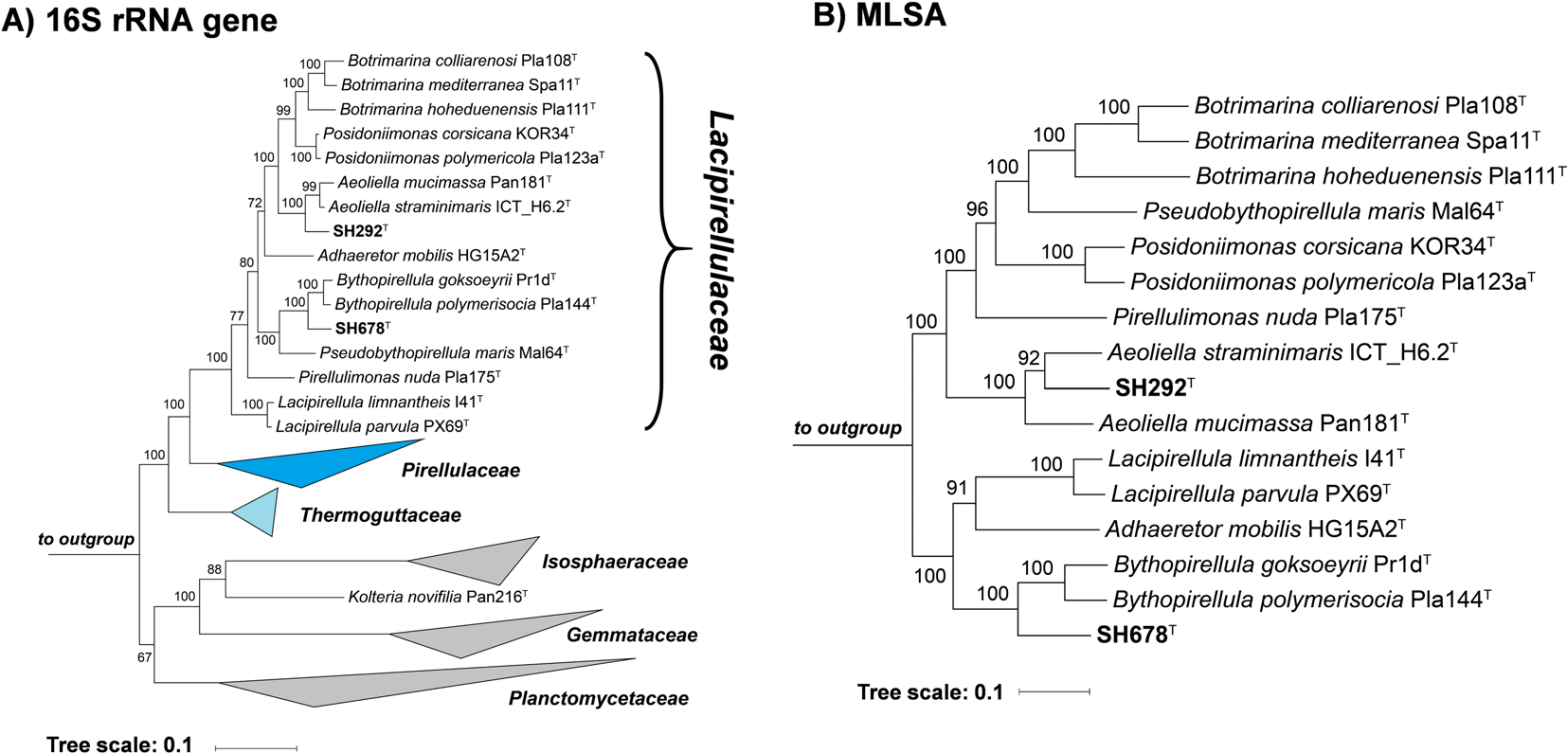
Phylogenetic trees. (**A**) Maximum likelihood phylogenetic tree based on 16S rRNA gene sequences showing the phylogenetic relationship of the novel isolates in the family *Lacipirellulaceae*. Bar, 0.1 substitutions per nucleotide position. (**B**) Multi-locus sequence analysis (MLSA)-based phylogenetic tree constructed with the genomes of characterized members in the family *Lacipirellulaceae*. The tree was computed based on a set of at least 30 single-copy gene-encoding proteins in a maximum likelihood approach with 1000 bootstrap replications. Bar, 0.1 substitution per amino acid position. Bootstrap values for both trees are given at the nodes (in %). Phylogenetic trees were visualized with iTOL v6.

**Fig. 2:**
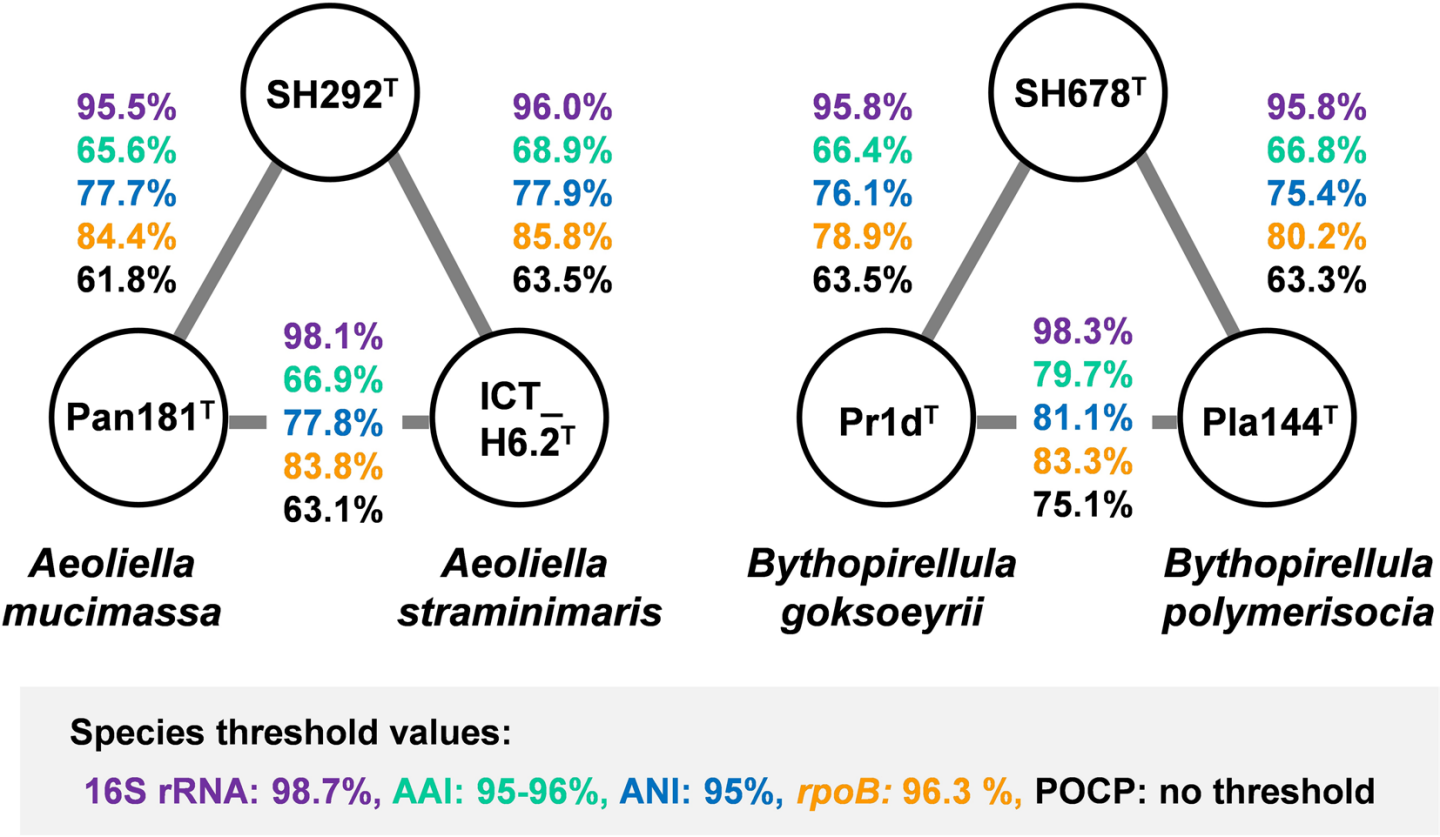
Comparison of phylogenetic markers for species delineation. Markers used: 16S rRNA gene sequence identity (16S rRNA), average amino acid identity (AAI), average nucleotide identity (ANI), sequence similarity of a partial sequence of the *rpoB* gene *(rpoB)*, percentage of conserved proteins (POCP). POCP values are typically used for the delineation of genera (genus threshold value of 50%, i.e. values > 50% indicate strains that belong to the same genus).

Nucleotide blast analyses of the 16S rRNA gene sequence of the novel isolates yielded similarity values in the range of 95–96% to *Aeoliella* spp. in case of strain SH292^T^ and *Bythopirellula* spp. in case of strain SH678^T^. Hence, the preliminary analysis placed both strains in the family *Lacipirellulaceae* which was subsequently confirmed by the clustering pattern in phylogenetic trees calculated based on 16S rRNA gene sequences and MLSA (Fig. 1). Both strains show 16S rRNA gene sequence similarity values below the commonly used species threshold of 98.7%^83^ when compared to their current closest neighbors. Thus, both likely belong to separate species of the described genera *Aeoliella* and *Bythopirellula*, respectively. Additional single gene- and genome-based phylogenetic markers were analyzed to support the delineation of the strains from described species. Indeed, the obtained values fell consistently below the species threshold values for ANI, AAI and partial *rpoB* sequence similarity (Fig. 2). A species threshold for POCP has not been proposed before; however, values in the range of 62–75% exclude that either of the two strains belongs to a novel genus (genus threshold of 50%)^78^. Taken together, all five analyzed markers are in line with positions of the two strains as members of novel species of the previously described genera^51,54^.

### Comparison of genomic features

The novel isolates have the smallest genomes and the highest DNA G + C content compared to the type strains of the current closest related species (Table 1). Their genome sizes are in the middle of the genome size range of the current family *Lacipirellulaceae* of 5.1–6.9 Mbp^84^. With the novel strains included, the DNA G + C content differs less than two percentage points within the two genera (58.0–60.0% for *Aeoliella* and 52.5–54.5% for *Bythopirellula*). The compared strains all harbour a single copy each of 5S, 16S and 23S rRNA genes and between 46 and 77 tRNA genes. For most of the other general features (number of protein-coding genes/Mbp, coding density, relative number of hypothetical proteins) only minor differences could be observed (Table 1). Strain SH678^T^ is likely to have extrachromosomal elements. Four linear sequences (with sizes of 1.2 to 29 kb) were obtained after genome assembly in addition to the circular chromosome. The 1.2 kb and the 6.9 kb sequence harbor putative transposase genes while the other two harbor (amongst others) genes encoding putative histidine kinases and phage proteins (integrase, terminase). No genes encoding putative (plasmid) replication initiator proteins (RPA) or proteins of a ParABS plasmid distribution system are encoded on these linear sequences. Genes encoding all three proteins, RPA, ParA and ParB, are frequently found on plasmids of planctomycetotal strains^85^, e.g. those belonging to the family *Isosphaeraceae* that consistently carry plasmids^12^. In this regard, it appears unlikely that the obtained linear sequences belong to an intact plasmid. The other analyzed strains lack extrachromosomal elements, however, for the two strains with only contig-level assemblies the information remains tentative (Table 1).

**Table 1:** Comparison of genomic features with the type strains of described species of the genera *Aeoliella* and *Bythopirellula*.

Characteristics	SH292^T^	*Aeoliella mucimassa* Pan181^T^	*Aeoliella straminimaris* ICT_H6.2^T^	SH678^T^	*Bythopirellula goksoeyrii* Pr1d^T^	*Bythopirellula polymerisocia* Pla144^T^
Genomic features						
Genome size (bp)	6,058,439	6,608,792	7,796,805	5,632,793	6,473,141	6,143,780
Contigs	1	1	98	5	8	28
Chromosome closed	Yes	Yes	No	Yes	Yes	No
Plasmids	No	No	Inconclusive	Yes (1–4)	No	Inconclusive
DNA G + C (%)	60.0	58.0	59.6	54.4	52.8	52.9
Completeness (%)	97.7	97.2	97.8	97.8	97.8	97.8
Contamination (%)	1.5	1.3	2.2	2.3	1.8	2.2
Genes	4744	5234	6058	4669	5148	4938
Genes/Mbp	783	792	777	829	795	804
Protein-coding genes	4680	5142	5948	4590	5034	4817
Protein-coding genes/Mbp	772	778	763	815	778	784
Hypothetical proteins*	1128	1253	1618	1127	1184	1162
Hypothetical proteins (%)	24.1	24.4	27.2	24.6	23.5	24.1
Coding density (%)	86.4	86.3	86.5	88.0	86.5	86.7
rRNA genes (5S,16S,23S)	1,1,1	1,1,1	1,1,1	1,1,1	1,1,1	1,1,1
tRNA genes	46	56	65	48	70	77
Secondary metabolite-associated biosynthetic gene clusters						
Terpene	2	3	3	3	2	2
Type I polyketide synthase	1	1	1	0	1	1
Type III polyketide synthase	1	1	1	1	0	0
NRPS-like	2	2	3	2	2	2
Ectoine	0	0	0	0	1	0
* N*-acetylglutaminyl-glutamine amide	0	0	1	0	1	1
Resorcinol	1	0	0	0	0	0
beta-lactone	0	1	1	0	1	1
Total number of BGCs	7	8	10	6	8	7
BGCs per Mbp	1.2	1.2	1.3	1.1	1.2	1.1
Carbohydrate-active enzymes						
Glycoside hydrolases	88	115	166	98	113	109
Glycosyltransferases	38	58	51	45	45	38
Polysaccharide lyases	5	10	21	15	19	22
Carbohydrate esterases	14	21	21	17	19	17
Carbohydrate-bind. modules	21	18	17	14	11	20
Auxiliary activities	2	1	2	1	4	2
CAZyme genes (total)	168	223	278	190	211	208
CAZyme genes per Mbp	28	34	36	34	33	34

Genome sequences of the strains used for comparison were downloaded from NCBI (under accession numbers mentioned in the effective species description articles)^51,52,54^. All six genomes were re-analysed with the same tools to ensure comparability.

*Based on the Refseq/PGAP-annotated genomes.

### Construction of the pangenome and analysis of genome-encoded functionalities

**Fig. 3:**
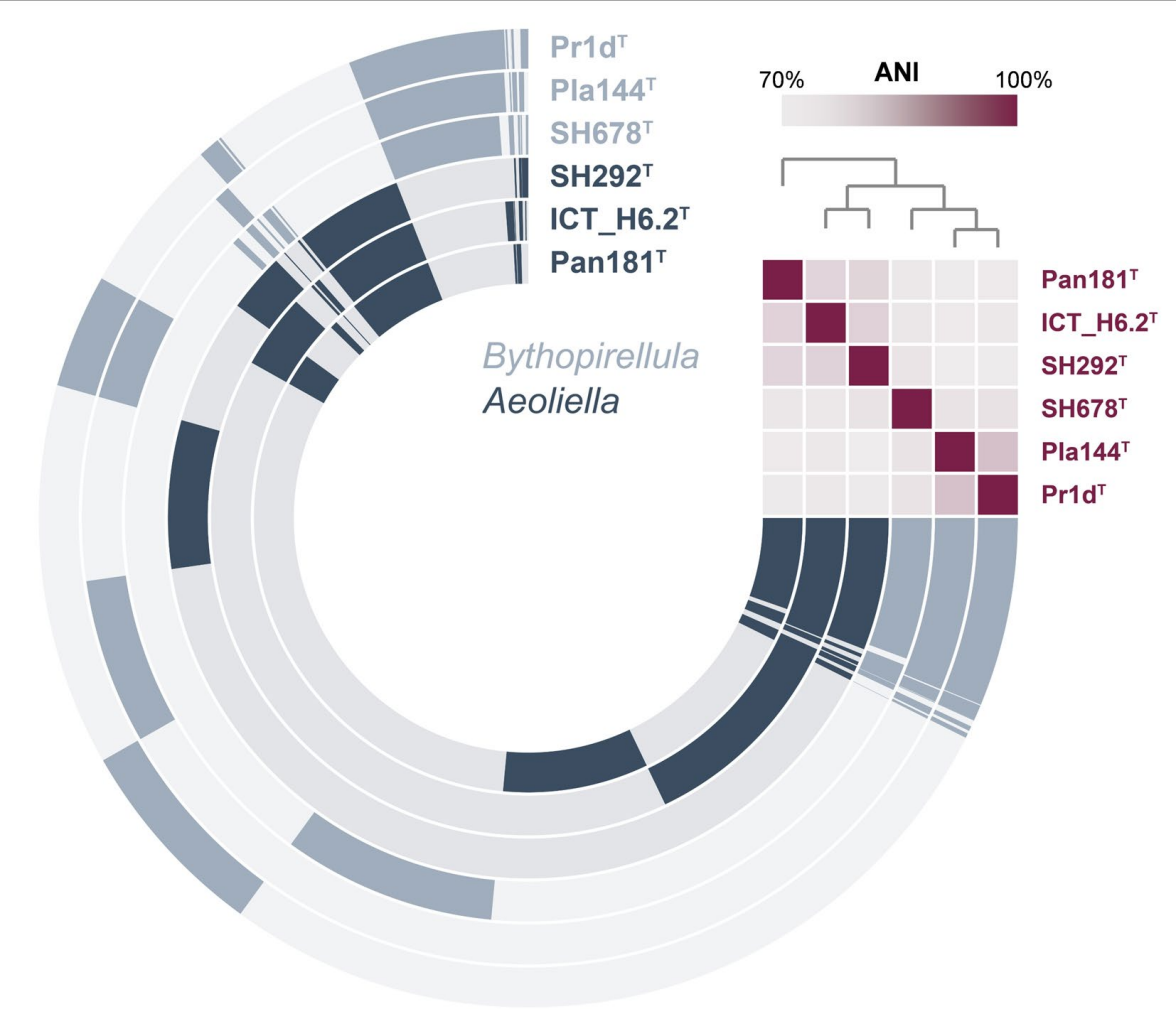
Pangenome reconstruction. Each open circle represents the pangenome of all genomes but is colored darker when the gene is present in the respective genome. The heatmap in the upper right corner indicates the degree of relationship based on ANI values (ANI ≤ 70%, pale bordeaux red to ANI = 100%, bright bordeaux red).

For visualizing the genome-based similarity, a pangenome was constructed based on the novel isolates and the characterized members of the genera *Aeoliella* and *Bythopirellula*. The obtained pangenome consisted of 15,890 gene clusters, of which 1,110 are conserved in all six genomes (core genome, 3 to 4 o’clock in the pangenome visualization) (Fig. 3). The intra-genus core genome contained 2,108 clusters for *Aeoliella* and 2,119 clusters for *Bythopirellula*. The intra-genus core genomes included the genes present in all compared genomes (3 o’clock in the pangenome visualization) and the accessory genes conserved within the genus members (11–12 o’clock in the pangenome visualization). The numbers of strain-specific clusters (singletons) accounted for 1,402 clusters in strain SH292^T^ and 1,780 clusters in strain SH678^T^. Of the exported singletons of strain SH292^T^, 573 could be assigned a COG accession and associated protein annotation (Table S3). The list includes proteins with various putative annotations/functions, e.g. transcriptional regulators, specialized sigma factors, adhesion factors, toxin-antitoxin systems, restriction modification systems, transporters, protein kinases or proteins involved in secretion. The curated list for strain SH678^T^ has 799 entries (Table S4) and revealed a high degree of overlapping putative annotations to the list obtained for strain SH292^T^. Several of the entries may thus represent homologous proteins with low sequence conservation.

The automated genome mining with antiSMASH resulted in 7–10 BGCs with potential relevance for secondary metabolite production per genome (Table 1). With 1.1–1.3 hits per Mbp, the number of predicted clusters is in the typical range for planctomycetal genomes^84^. A resorcinol biosynthesis-related cluster is specific for strain SH292^T^, whereas strain SH678^T^ harbors a putative type III polyketide synthase gene that is not present in the other two *Bythopirellula* species. The type III polyketide synthase cluster in strain SH292^T^ consists of a potential three-gene operon encoding a putative methyltransferase, an oxidoreductase and a type III polyketide synthase. The encoded proteins have previously been linked to alkylresorcinol biosynthesis in other planctomycetal families^42^. The other clusters identified in the novel isolates have not been associated to the biosynthesis of specific compounds yet. Enzymes encoded by terpenoid biosynthetic genes are likely not relevant for carotenoid biosynthesis since both strains are non-pigmented.

An analysis for CAZyme-encoding genes yielded between 168 and 278 hits for the analyzed genomes (Table 1). In each genome, ca. 50% of the putative CAZymes belong to the class of glycoside hydrolases and 20–30% to the class of glycosyltransferases. With 33–36 CAZyme-encoding genes per Mbp, the numbers are consistent for the compared genomes, except for strain SH292^T^ that only harbors 28 putative CAZyme genes per Mbp. The strain has a reduced CAZyme content for most enzyme classes (especially polysaccharide lyases) but is enriched in proteins with carbohydrate-binding modules (CBMs).

### Morphological and physiological features

**Fig. 4:**
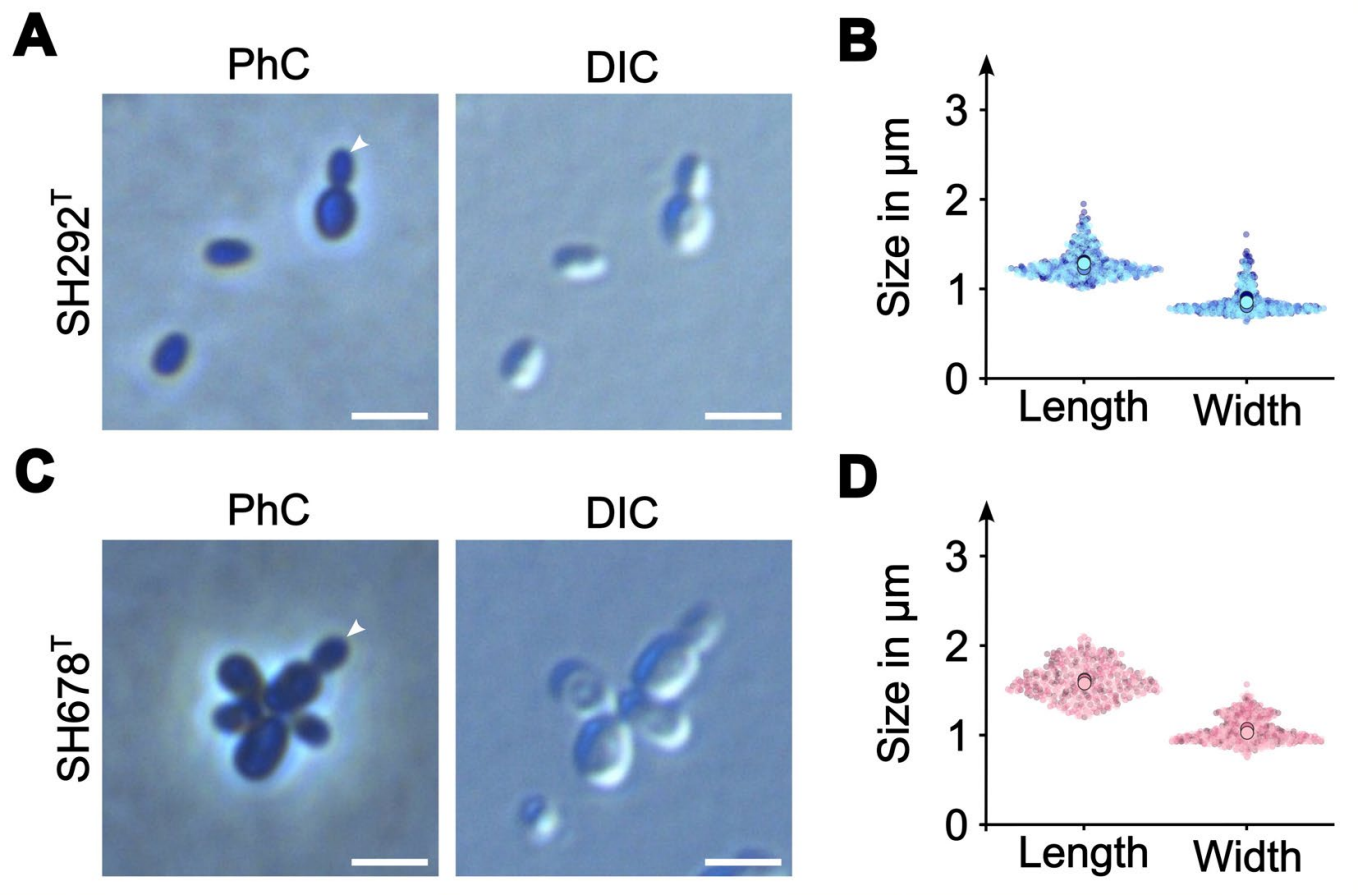
Cell morphology and cell size of strains SH292^T^ and SH678^T^. (**A**) and (**C**) Microscopic images of strains SH292^T^ and SH678^T^ using Phase contrast (PhC) and differential interference contrast (DIC). Dividing cells are marked with an arrow head. (**B**) and (**D**) Cell size analyses of strain SH292^T^, and SH678^T^. Mean values are depicted as larger circles. Scale bars represent 2 µm.

**Fig. 5:**
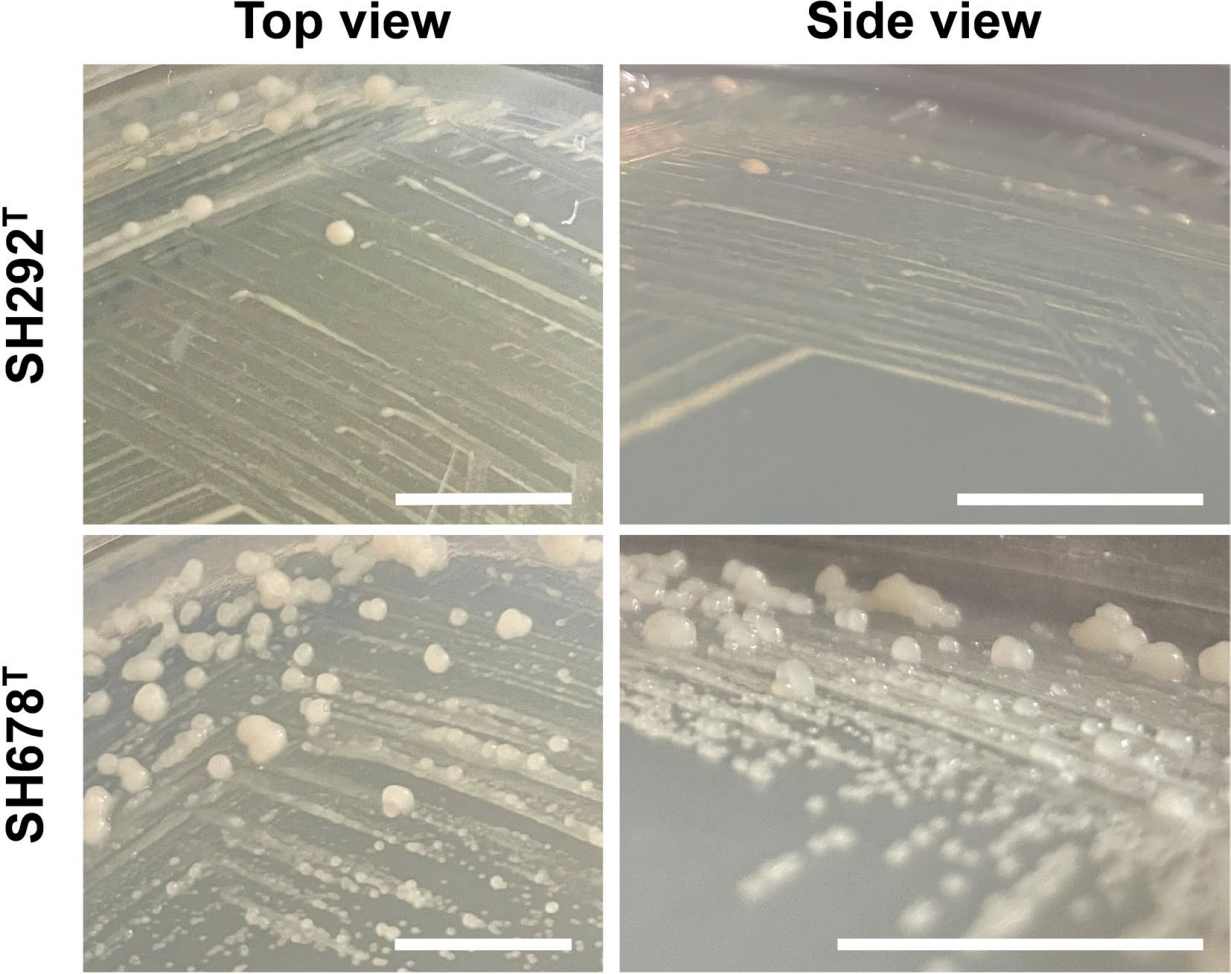
Appearance of colonies on plates. Colonies of the two isolates on agar plates after cultivation under optimal temperature and pH conditions for five weeks. The scale bars represent 1 cm.

Cells of strain SH292^T^ are pear-shaped and cells of strain SH678^T^ are oval to pear-shaped (Fig. 4A,C). The cells have a mean cell length and width of 1.3 ± 0.2 μm × 0.9 ± 0.1 μm (SH292^T^) and 1.6 ± 0.2 μm × 1.1 ± 0.1 μm (SH678^T^) (Fig. 4B,D). Strain SH292^T^ does not form microscopical aggregates, an ability separating this strain from its closest relative *Aeoliella straminimaris* ICT_H6.2^T^. In contrast, cells of strain SH678^T^ can form microscopical shapeless aggregates and rosettes. Its current closest relative *Bythopirellula polymerisocia* Poly144^T^ forms fibrous aggregates too. Asymmetric cell division via polar budding is followed by both novel strains (Fig. 4A,C) in a similar fashion as observed for the described members of the family. Colonies of the novel isolates are white to cream-colored which is in line with the colony color of the close relatives. Colonies of strain SH292^T^ are circular and raised to convex, whereas strain SH678^T^ forms more irregular-shaped pulvinate colonies (Fig. 5). While the already described type strains of species belonging to the genera *Aeoliella* and *Bythopirellula* have been isolated from marine or brackish habitats, the novel isolates SH292^T^ and SH678^T^ were isolated from a limnic to slightly brackish habitat. Like the previously described species, the novel isolates are aerobic, mesophilic and neutrophilic heterotrophs (Table 2). The determined temperature optimum for growth falls between 25 and 28 °C for all compared strains. However, the type strains of the so far described *Aeoliella* species are the only strains that grow at temperatures exceeding 30 °C and even show growth at 37–39 °C.

**Table 2:** Comparison of phenotypic characteristics of the novel isolates.

Characteristics	SH292^T^	*Aeoliella mucimassa* Pan181^T^	*Aeoliella straminimaris* ICT_H6.2^T^	SH678^T^	*Bythopirellula goksoeyrii* Pr1d^T^	*Bythopirellula polymerisocia* Pla144^T^
Sampling information						
Location	Lake Fuhlensee, Strande, Germany	Tyrrhenian Sea close to Panarea Island, Italy	Tagus river estuary (brackish), Portugal	Lake Fuhlensee, Strande, Germany	Mohns Ridge (part of Arctic Mid Ocean Ridge), Arctic Ocean	Eastuary of the Baltic Sea, Germany
Sampled Material	Surface water	Yellow-gray fringes	Sediment samples	Surface water	Iron hydroxide deposits (600 m depth)	Plastic waste (polyethylene)
Phenotypic features						
Pigmentation	White to cream	White	White to beige	White to cream	White	White
Cell Shape	Pear-shaped	Pear-shaped	Spherical to ovoid	Oval to pear-shaped	Spherical to ovoid	Pear-shaped
Size (length x width) (µm)	1.3 ± 0.2 × 0.9 ± 0.1	1.4 ± 0.3 × 0.7 ± 0.1	1.4 × 1.1	1.6 ± 0.2 × 1.1 ± 0.1	0.5–1.5	1.1 ± 0.1 × 0.7 ± 0.1
Cell Division Mode	Budding	Budding	Budding	Budding	Budding	Budding
Temperature Range (optimum) (°C)	18–28 (25)	10–39 (28)	10–37 (25)	18–28 (25)	10–27 (25)	20–30 (27)
pH Range (optimum)	6.0–9.0 (7.5)	5.5–9.0 (7.5)	6.5–10.0 (7.5)	6.0–8.0 (7.0)	n.d.	6.0–9.5 (8.5)
Relation to Oxygen	Aerobic	Aerobic	Aerobic, grows under microaerobic conditions	Aerobic	Aerobic	Aerobic
Aggregates	No	Yes, very fibrous	Yes	Yes, shapeless aggregates or rosettes	Yes	Yes

Data on the strains used for comparison was taken from the effective species description articles^51,52,54^. Since the compared strains were grown in different media, inter-strain comparisons are medium-dependent. For strains SH292^T^ and SH678^T^, the temperature optimum for growth was determined by evaluating fastest growth on agar plates, whereas growth rates determined in liquid cultures were used for determination of the respective pH optimum for growth.

n.d., not determined.

### Ecological context

To get information on the ecological context of close relatives of the novel isolates that belong to the same species, additional analyses were performed. The classification of the two strains based on the GTDB taxonomy gave “*Aeoliella* sp024054565” as output for strain SH292 and “*Bythopirellula goksoeyrii*” for strain SH678. The species placeholder for strain SH292 was removed from the GTDB and a reference genome is no longer assigned to this taxon placeholder. For “*Bythopirellula goksoeyrii*” the GTDB lists only the type strain Pr1d that was already considered for comparison. Since the GTDB did not allow any conclusions on the ecological context, the 16S rRNA gene sequences were used for nucleotide BLAST analysis against the Core nucleotide database (core_nt) of NCBI. The analysis yielded six environmental sequences related to the 16S rRNA gene of strain SH292^T^ and three related to SH678^T^ (Table S5). Strains belonging to the same species as strain SH292^T^ have been detected in terrestrial habitats, e.g. the gut of the earthworm *Eisenia fetida*, waste sites and soil. Close relatives of strain SH678^T^ were found in aquatic or terrestrial systems including a sulfidic spring, a water purification system and a gold mine.

## Conclusion

Based on the collected data, we conclude that strain SH292^T^ belongs to a novel species in the genus *Aeoliella*, whereas strain SH678^T^ belongs to a novel species of the genus *Bythopirellula*.

### Description of *Aeoliella fuhlenseeensis* sp. nov.

fuh.len.see.en’sis. N.L. fem. adj. *fuhlenseeensis*, pertaining to Lake Fuhlensee, from which the type strain was isolated.

Aerobic, mesophilic and neutrophilic heterotroph. Cells are pear-shaped, have an average size of 1.3 ± 0.2 μm × 0.9 ± 0.1 μm and divide asymmetrically by polar budding. Cells form white to cream-colored, circular and raised to convex colonies. The type strain is SH292^T^ (= DSM 116587^T^ = KCTC 102092^T^). It was isolated from surface water of Lake Fuhlensee, Strande, Schleswig–Holstein, Germany. The type strain has a genome size of 6.06 Mbp with a DNA G + C content of 60.0% and lacks extrachromosomal elements. Growth of the type strain was observed from 18 to 28 °C and pH 6.0–9.0 with optimal growth at 25 °C and pH 7.5.

### *Description of Bythopirellula superficialaquae* sp. nov.

su.per.fi.ci.al.a'quae. L. masc. adj. *superficialis*, superficial; L. fem. n. *aqua*, water; N.L. gen. n. *superficialaquae*, of surface water, reflecting the sampling material from which the type strain was isolated.

Aerobic, mesophilic and neutrophilic heterotroph. Cells are oval to pear-shaped, have an average size of 1.6 ± 0.2 μm × 1.1 ± 0.1 μm and divide asymmetrically by polar budding. Cells form white to cream-colored and round to irregular-shaped pulvinate colonies. The type strain is SH678^T^ (= DSM 116729^T^ = KCTC 102079^T^). It was isolated from surface water of Lake Fuhlensee, Strande, Schleswig–Holstein, Germany. The type strain has a genome size of 5.63 Mbp with a DNA G + C content of 52.8%. Extrachromosomal elements are present. Growth of the type strain was observed from 18 to 28 °C and pH 6.0–8.0 with optimal growth at 25 °C and pH 7.0.

## Supplementary Information

Below is the link to the electronic supplementary material.

Supplementary Material 1

Supplementary Material 2

Supplementary Material 3

Supplementary Material 4

Supplementary Material 5

## Data Availability

The 16S rRNA gene sequences were deposited in the GenBank database under the accession numbers PV984964 (SH292^T^) and PV984966 (SH678^T^). Genome sequence information is available from NCBI under the following accession numbers: CP197417 (SH292^T^), CP197426 (SH678^T^, chromosome), CP197427 (plasmid pSH678_1), CP197428 (plasmid pSH678_2), CP197429 (plasmid pSH678_3), and CP197430 (plasmid pSH678_4).
